# Molecular characterization and secreted production of basidiomycetous cell-bound β-glycosidases applicable to production of galactooligosaccharides

**DOI:** 10.1093/jimb/kuab087

**Published:** 2021-12-08

**Authors:** Eiji Ishikawa, Masakazu Ikeda, Hidetsugu Sotoya, Minako Anbe, Hoshitaka Matsumoto, Mayumi Kiwaki, Hiroshi Hatano

**Affiliations:** Yakult Central Institute, 5-11 Izumi, Kunitachi-shi, Tokyo, 186-8650, Japan; Yakult Pharmaceutical Industry Co., Ltd, 5-11 Izumi, Kunitachi-shi, Tokyo, 186-8650, Japan; Yakult Central Institute, 5-11 Izumi, Kunitachi-shi, Tokyo, 186-8650, Japan; Yakult Central Institute, 5-11 Izumi, Kunitachi-shi, Tokyo, 186-8650, Japan; Yakult Central Institute, 5-11 Izumi, Kunitachi-shi, Tokyo, 186-8650, Japan; Yakult Central Institute, 5-11 Izumi, Kunitachi-shi, Tokyo, 186-8650, Japan; Yakult Central Institute, 5-11 Izumi, Kunitachi-shi, Tokyo, 186-8650, Japan

**Keywords:** soluble protein, glycoside hydrolase family 1, thermostability, GOS isomer, enzyme preparation

## Abstract

Cell-bound β-glycosidases of basidiomycetous yeasts show promise as biocatalysts in galactooligosaccharide (GOS) production. Using degenerated primers designed from *Hamamotoa singularis* (Hs) *bglA* gene, we newly identified three genes that encode cell-bound β-glycosidase from *Sirobasidium magnum* (Sm), *Rhodotorula minuta* (Rm), and *Sterigmatomyces elviae* (Se). These three genes, also named *bglA*, encoded family 1 glycosyl hydrolases with molecular masses of 67‒77 kDa. The BglA enzymes were approximately 44% identical to the Hs-BglA enzyme and possessed a unique domain at the N-terminus comprising 110 or 210 amino acids. The Sm-, Rm-, and Se-BglA enzymes as well as the Hs-BglA enzyme were successfully produced by recombinant *Aspergillus oryzae*, and all enzymes were entirely secreted to the supernatants. Furthermore, addition of some nonionic detergents (e.g. 0.4% [v/v] Triton-X) increased the production, especially of the Hs- or Se-BglA enzyme. Out of the BglA enzymes, the Se-BglA enzyme showed remarkable thermostability (∼70°C). Additionally, the Sm- and Se-BglA enzymes had better GOS yields, so there was less residual lactose than in others. Accordingly, the basidiomycetous BglA enzymes produced by recombinant *A. oryzae* would be applicable to GOS production, and the Se-BglA enzyme appeared to be the most promising enzyme for industrial uses.

## Introduction

Galactooligosaccharides (GOS) are non-digestible carbohydrates that are resistant to gastrointestinal digestive enzymes but are fermented by bifidobacteria, which results in activation of these bacteria within the intestinal flora (Ohtsuka, 1989; Tanaka et al., 1983). Thus, GOS are being considered as prebiotic food ingredients for a variety of foods (Sako et al., 1999).

GOS are produced commercially from lactose by the transgalactosidase activity of microbial β-galactosidases. Many β-galactosidases that produce GOS have been isolated and purified from bacteria (Huber et al., 1976; Mozaffar et al., 1984) or fungi (Asp et al., 1980; Maugard et al., 2003; Toba et al., 1985), and basidiomycetous cell-bound glycoside hydrolases have received particular attention for their high transgalactosidase activity (Blakely & MacKenzie, 1969; Cho et al., 2003; Ohtsuka et al., 1990; Onishi & Tanaka, 1995, 1996, 1998; Onishi et al., 1995, 1996). We previously developed a *Hamamotoa* (formerly *Sporobolomyces*) *singularis* (Hs) cell-bound β-glycosidase (also called β-galactosidase-like enzyme or β-hexosyl transferase) for industrial GOS production (Ishikawa et al., 2005; Sakai et al., 2008).

However, because cell-bound β-glycosidase is difficult to release from Hs cells, the microbial conversion process involves using a concentrate of the cells, which raises certain issues in the industrial process for GOS such as (i) impurities that increase costs later in the GOS production process, (ii) a concentrate stronger than a cell cake is impossible to make, and (iii) cell cakes are bulky making them difficult to ship and handle. Although researchers tried using recombinant *Pichia pastoris* (Dagher et al., 2013) for secreted production of the cell-bound β-glycosidase, the amount of secreted enzyme did not meet industrial requirements.

Therefore, we aimed to develop soluble enzyme preparations of basidiomycetous cell-bound β-glycosidases that would be applicable to GOS production. This paper describes (i) gene cloning of the cell-bound β-glycosidases from three basidiomycetous yeasts related to Hs, (ii) secreted production of cell-bound β-glycosidases using *Aspergillus oryzae* as a host, (iii) evaluation of the recombinant β-glycosidases as catalysts for GOS production, and (iv) characterization of the GOS produced from the recombinant β-glycosidases. These results provide several seeds for developing novel enzyme preparations, industrial GOS processes, or both.

## Materials and Methods

### Microbial Strains and Culture Conditions

*Hamamotoa* (formerly *Sporobolomyces*) *singularis* ATCC 24193 was obtained from the American Type Culture Collection (Manassas, Virginia, USA). *Rhodotorula minuta* (Rm) CBS 319 was obtained from the CBS-KNAW culture collection (Utrecht, The Netherlands). *Sirobasidium magnum* (Sm) JCM 6876 was obtained from the Japan Collection of Microorganisms (Ibaraki, Japan). *Sterigmatomyces elviae* (Se) IFO 1843 was obtained from the Institute for Fermentation (Osaka, Japan). The yeasts, resuspended in 10% glycerol, were stored at –80°C and were cultivated in a medium that contained 10 g yeast extract (Difco Laboratories, Detroit, MI, USA), 1 g KH_2_PO_4_, 0.5 g MgSO_4_·7H_2_0, and 50 g of lactose or glucose in 1.0 L distilled water (pH 5.0). Glycerol stocks were inoculated into 150 mL of fresh medium in a 500-mL baffled shake flask stoppered with a cotton plug and incubated at 27°C on a rotary shaker (180 rpm) to give a pre-culture. Then a 5-mL portion of the day-three pre-culture, which was in the late exponential phase (OD_660 nm_ = 10; 4 × 10^8^ cells/mL), was inoculated and cultivated for seven days under conditions identical to that of the pre-culture.

### Cloning *bglA* Genes From Basidiomycetous Yeasts

We harvested cells in the exponential growth phase (OD_660 nm_ = 5; 2 × 10^8^ cells/mL) and resuspended them in 50 mM phosphate citrate buffer (pH 4.0), added 1% (wt/vol) of Usukizyme (Kyowa Chemical Products, Osaka, Japan) in the same buffer, and incubated the mixture at 37°C for 1 h to degrade the cell walls. We extracted ribonucleic acid (RNA) and deoxyribonucleic acid (DNA) with Trizol (Thermo Fisher Scientific, Waltham, MA, USA) and ZR Fungal/Bacterial DNA MiniPrep (Zymo Research, CA, USA), respectively. Total RNA was treated with RNase-free DNase I (Takara Bio, Shiga, Japan) and reverse transcribed with oligo-(dT)20 and PrimeScript® 1st strand cDNA Synthesis Kit (Takara Bio).

Employing the 1st strand cDNAs as templates, we performed polymerase chain reactions (PCRs) with degenerated primers (Table 1) and rapid amplification cDNA end (RACE) using Tks Gflex™ DNA Polymerase (Takara Bio) and 5'/3' RACE kit, 2nd generation (Roche Diagnostics, Tokyo, Japan), respectively. Inverse PCR (IPCR) was carried out as previously described (Ishikawa et al., 2005). We digested 500 ng of genomic DNA with *Hind*III, diluted it, and subjected it to self-ligation with T4 DNA ligase (New England Biolabs, Ipswich, MA, USA). With these ligation mixtures as templates, amplifications were carried out with the specific primers designed from the internal sequence.

**Table 1.** Degenerated Primers Used for *bglA* cDNA Cloning

Name	Amino acid sequence	Direction	Nucleotide sequence^a^
F1	AGAAIQVEGA	Forward	GCCGGCGCGGCTATHCARGTNGARGGNGCN
F2	VKTWFTFNEP	Forward	GTCAAGACNTGGTTYACNTTYAAYGARCCN
R1	IYFSEFGWAE	Reverse	CTCGGCCCACCCRAAYTCNSWRAARTADAT
R2	WSFVDNWEW	Reverse	CCATTCCCARTTRTCNACRAANSWCCA
C-R70	DNFEWNTGLV	Reverse	GACGAGGCCNSWRTTCCAYTCRAARTTRTC

^a^M (A, C); R (A, G); W (A, T); S (C, G); Y (C, T); K (G, T); V (A, C, G); H (A, C, T); D (A, G, T), B (C, G, T); N (A, C, G, T) are used to represent mixed-base nucleotides.

### Nucleotide Sequencing

Nucleotide sequences were determined with an ABI PRISM 3130 *xl* DNA sequencer and BigDye Terminator version 3.1 (Thermo Fisher Scientific) and assembled with ATGC bundled with Genetyx version 14.0 (Genetyx, Tokyo, Japan).

Next-generation sequencing (NGS) was also used for collecting supportive data. Sequence libraries were prepared using a TruSeq DNA PCR-Free Sample Prep LS Kit (Illumina, San Diego, CA, USA) and DNA Shearing System M220 (Covaris, Woburn, MA, USA) following the manufacturers’ instructions. The prepared sequence library was subjected to 250 bp × 2 paired end sequencing by MiSeq (Illumina) to determine the nucleotide sequence, which was then imported into CLC Genomics Workbench (QIAGEN, Aarhus, Denmark) to assemble draft genomes.

The nucleotide sequences of genes, cDNAs encoding the β-glycosidases (*bglA*s), and codon-optimized cDNAs were submitted to the DDBJ/GENBANK/EMBL nucleotide databanks under accession numbers LC597225 (*Sirobasidium magnum bglA* gene), LC597226 (*Rhodotorula minuta bglA* gene), LC597227 (S*terigmatomyces elviae bglA* gene), LC597228 (*Hamamotoa singularis* codon-optimized *bglA* cDNA), LC597229 (*Sirobasidium magnum* codon-optimized *bglA* cDNA), LC597230 (*Rhodotorula minuta* codon-optimized *bglA* cDNA), and LC597231 (S*terigmatomyces elviae* codon-optimized *bglA* cDNA). The amino acid sequences of the BglA enzymes were deduced from the nucleotide sequence of the cDNA. The N-terminal methionine corresponding to the initiation codon was numbered 1, and the amino acid sequences were aligned with the N-terminus on the left and the C-terminus on the right. Amino acid numbers are designated by a superscript (e.g. K^210^).

### Production of Recombinant Proteins

As for the *Escherichia coli* system, we used the pCold system (Takara Bio). In the case of *Saccharomyces cerevisiae* and *P. pastoris*, we used pYES2 Yeast Expression Vector (Thermo Fisher Scientific, Waltham, MA, USA) and pPICZα A, B, & C *Pichia* Vectors (Thermo Fisher Scientific), respectively. As for the *A. oryzae* system, we used an outsourcing service provided by Ozeki Corporation (Hyogo, Japan) (Koda et al., 2004; Tsuboi et al., 2005) (https://www.ozeki.co.jp/food_bio/protein_expression/aspergillus.html). The *bglA* cDNAs were synthesized based on a codon usage of *A. oryzae*, and the signal peptide of each *bglA* cDNA was replaced with the TAA signal peptide of *A. oryzae* Taka-amylase (Tsukagoshi et al., 1989).

### Effects of Temperature or pH on Enzyme Activities

β-Glycosidase activity was measured by determining the amount of hydrolysis of *o*-nitrophenyl-β-d-galactopyranoside (ONPG) (Kanto Kagaku, Tokyo, Japan). The incubation mixture contained 10 mM ONPG, 50 mM phosphate citrate buffer (pH 4.0), and the crude enzyme in a total volume of 1 mL. The standard reaction was carried out at 30°C for 10 min and then stopped by adding 4 mL of 0.25 M Na_2_CO_3_. Absorbance at 420 nm was determined for each supernatant as a measure of the amount of *o*-nitro-phenol (ONP) released. One unit (U) of β-glycosidase was defined as the amount of enzyme that hydrolyzed 1 μmol of ONPG per minute under the assay conditions. The assay was repeated three times for every sample, and the results were averaged. The pH dependency was determined in 1/10 McIlvaine buffer (pH 3‒8) instead of the standard condition of 50 mM phosphate citrate buffer (pH 4.0) mentioned above. As for thermal stabilities, the 30 μL of crude enzymes were heated by a thermal cycler (C1000 Touch™ Thermal Cycler, Bio-Rad, Hercules, CA, USA) for 30 min before the assay, and then the residual activities were measured.

### Preparation of Crude Enzyme

Recombinant *A. oryzae* was aerobically cultured in dextrin-peptone-yeast extract (DPY) medium comprising 20 g dextrin (Sigma-Aldrich, St. Louis, MO, USA), 10 g polypeptone (Difco Laboratories), 5 g yeast extract (Difco Laboratories), 5 g KH_2_PO_4_, and 0.5 g MgSO_4_·7H_2_0 in 1.0 L distilled water (pH 5.5). Each detergent was added to the DPY medium before autoclave. A loop of recombinant *A. oryzae* was inoculated to 150 mL of DPY medium in a 500-mL baffled shake flask stoppered with a cotton plug and incubated at 30°C on a rotary shaker (180 rpm) for 168 h.

The recombinant *A. oryzae* culture was filtered through No. 5A filter paper (ADVANTEC, Tokyo, Japan) followed by 0.45 μm Millipore Durapore® (Merck Millipore, Burlington, MA, USA). Then the filtrate was concentrated by using Amicon® Ultra-15 Centrifugal Filter Units (Merck Millipore).

### Bioconversion of Lactose to GOS

The incubation mixture (containing 66.7 g lactose and crude enzyme equivalent to 8.0 U of β-glycosidase in a total volume of 100 mL) was adjusted to pH 6.0 with Na_2_CO_3_, incubated at 64°C, and the reaction was stopped by boiling for 5 min. It was possible to incubate the reaction mixture at a higher temperature than that used in the enzyme assay because the high sugar concentration of the reaction mixture (66.7% (w/v)) is protective for BglA enzymes against heat denaturation. The amount of GOS formed in the reaction mixture (i.e. degree of polymerization) was monitored by high-performance liquid chromatography (HPLC) with equipment that included a reflective integrator (Shodex RI-101, Showa-Denko, Tokyo, Japan). The mobile phase was distilled water and the column was a Shodex SUGAR KS-802 (8 × 300 mm, Showa-Denko). The data acquisition and calculation of area ratio were carried out by the Waters Empower 2 Software (Waters Corporation, Milford, MA, USA).

### GOS Isomer Analysis

Each GOS sample was separated into four fractions—disaccharides, trisaccharides, tetrasaccharides, and penta- or higher-saccharides—by open-column chromatography with Bio-gel P-2 (80 mm × 100 cm; Bio-Rad Laboratories, Hercules, CA, USA) for subsequent analysis. To separate these fractions definitely and to avoid cross-contamination, GOS was converted into pyridylamino derivatives after gel filtration and subjected to HPLC, as explained in previous reports (Kimura et al., 1995; Yanahira et al., 1995).

## Results

### Cloning of *bglA* Genes From Three Basidiomycetous Yeasts

**Fig. 1.**
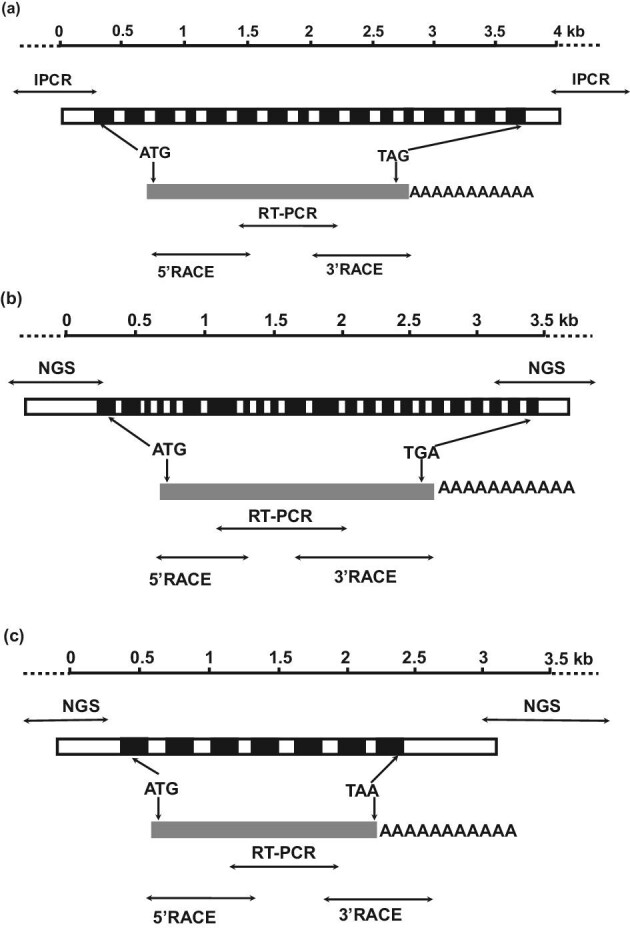
Structures of basidiomycetous *bglA* genes and mRNAs. (a) *Sirobasidium magnum* (Sm). (b) *Rhodotorula minuta* (Rm). (c) *Sterigmatomyces elviae* (Se). Exons, indicated by black boxes, were determined from a comparison with cDNA, indicated by a gray box. Reverse transcription-PCR was carried out with degenerated primers. Full-length cDNA was obtained by 5’ and 3’ RACE. 5’ and 3’ untranscribed regions of the *bglA* genes were cloned by IPCR or NGS. ATG and TGA/TAG/TAA are the initiation and stop codons, respectively, for protein synthesis.

Amino acid sequences of the Hs-BglA enzyme (Ishikawa et al., 2005) and plant β-glucosidases similar to the Hs-BglA enzyme were subjected to multiple sequence alignment by ClustalW2. Based on the amino acid sequences of putatively conserved regions, degenerated primers were designed (Table 1). Using the degenerated primers F2 and R2 with Hs-DNA as a positive control, we could identify specific amplicons that were almost identical to Hs in size in Rm and Se, but not in Sm (Supplementary Fig. S1). Using another primer set with F1 and C-R70, we could identify the specific amplicon in Sm. Using the internal sequences of these amplicons, full length cDNAs were identified by 3’ and 5’ RACE. Furthermore, 3’ and 5’ untranscribed regions of these genes were identified by IPCR and NGS (Fig. 1). The nucleotide sequences of genes, that is, the cDNAs encoding the cell-bound β-glycosidases, were also named “*bglA*” and submitted to the DDBJ/GENBANK/EMBL nucleotide databanks under accession numbers LC597225, LC597226, and LC597227.

### Comparison of BglA Enzymes

The Sm-, Rm-, Se-, and Hs-BglA enzymes were 43%–60% similar to one another (Supplementary Fig. S1). The features of each BglA enzyme based on its deduced amino acid sequence are summarized in Table 2. The molecular masses of the BglA enzymes calculated from the amino acid sequences ranged from 65 to 77 KDa, with the Sm-BglA enzyme being the largest. Putative N-glycosylation sites were identified, and these suggest that the native enzymes are glycosylated, which is consistent with previous reports (Onishi & Tanaka, 1995, 1996).

**Table 2.** Comparison of Basidiomycetous BglA Enzymes

	*Hamamotoa singularis* ^a^	*Sirobasidium magnum*	*Rhodotorula minuta*	*Sterigmatomyces elviae*
Calculated molecular mass^b^	65,620	76,513	67,356	66,612
Amino acids^b^	594	701	600	594
Isoelectric point^b^	4.98	4.4	5.25	4.58
Putative N-glycosylation sites^c^	4	9	7	7
Glycoside hydrolase family	GH1	GH1	GH1	GH1
Unique N-terminal domain^d^	110	210	110	110
Signal peptide cleavage site(s)	19/20 and/or 22/23^e^	16/17^f^	19/20^f^	19/20^f^
Putative catalytic centers^g^	E^275^, E^496^	E^385^, E^600^	E^279^, E^501^	E^271^, E^495^

^a^Ishikawa et al. (2005).

^b^Including signal peptide.

^c^The motif NX[S/T] was searched for in the deduced amino acid sequence.

^d^Length of amino acid sequence.

^e^Determined by Edman degradation.

^f^Predicted by Genetyx ver. 14.0.

^g^Predicted by ClustalW2, the superscript represents the amino acid number from N-terminal methionine.

The main modules of the basidiomycetous BglA enzymes are family 1 glycosyl hydrolases (GH1) (Henrissat & Bairoch, 1993). The GH1 modules comprised approximately 500 amino acids and were similar to plant β-glucosidases, but all the BglA enzymes presented here possessed a unique N-terminal domain comprising 110 or 210 amino acids, which accounts for their differences in molecular mass. Putative signal peptides of approximately 20 hydrophobic amino acids were predicted in all the BglA enzymes, and the N-termini of mature Hs-BglA enzyme were confirmed by Edman degradation (Table 2) (Ishikawa et al., 2005). Multiple sequence alignment predicted that the BglA enzymes possess putative catalytic centers comprising two glutamate residues: one with a typical TFNEP motif and the other with a deviant ITENG motif (F^494^SEFG in Hs-BglA, L^598^SEFG in Sm-BglA, L^499^TEFG in Rm-BglA, and I^493^SEFG in Se-BglA, with the superscript representing the amino acid number from N-terminal methionine) (Supplementary Fig. S1), both of which are generally conserved as catalytic centers in plant β-glucosidases belonging to GH1 (Zhou et al., 2002). The phylogenic tree based on amino acid sequences revealed that the BglA enzymes and some other fungal β-glucosidases possessing unique N-terminal domains formed a cluster that was relatively distant from plant β-glucosidases, which do not possess these unique N-terminal domains (Supplementary Fig. S2).

### Comparison of *bglA* Genes

The features of each *bglA* gene are summarized in Table 3. Many introns were identified in all the *bglA* genes, but the Se-*bglA* gene had relatively fewer introns, as is reflected in its notably higher exon/intron ratio. Consistent with the number of introns, several microexons were identified in *bglA* genes other than the Se-*bglA* gene. The length of introns in all the *bglA* genes was similar, whereas the mean length of exons varied on each yeast. Notably, the 11th intron in the Rm-*bglA* gene did not fulfil the “GT-AG” rule because it had “GC-AG” borders flanking the microexons, which is a characteristic also seen in the 5th intron in the Hs-*bglA* gene.

**Table 3.** Comparison of Basidiomycetous *bglA* Genes

		*Hamamotoa singularis* ^a^	*Sirobasidium magnum*	*Rhodotorula minuta*	*Sterigmatomyces elviae*
Transcribed region (bp)		3047	3605	3347	2328
Exon	Number	19	17	22	7
	Max. (bp)	220	586	414	527
	Min. (bp)	18	5	6	64
	Mean ± SD (bp)	94 ± 66	136 ± 135	95 ± 108	281 ± 168
	Number of microexons^b^	3	2	10	0
Intron	Number	18	16	21	6
	Max. (bp)	86	139	69	67
	Min. (bp)	50	57	55	54
	Mean ± SD (bp)	59 ± 8	81 ± 20	60 ± 4	60 ± 5
	Not fulfilling “GT-AG” rule^c^	1	0	1	0
Exon/intron ratio^d^		1.68	1.78	1.64	5.43

^a^Ishikawa et al. (2005).

^b^Less than 30 bp.

^c^The number of border sequences between the exons and introns did not fulfill the ‘GT-AG’ rule.

^d^Calculated based on the mRNA and the relevant region of *bglA* gene.

### Production of Recombinant BglA Enzymes

Initially, we tried to produce recombinant BglA enzymes using an *E. coli* expression system and native cDNAs, but inclusion bodies prevented capture of active BglA enzymes. Furthermore, enzyme activity was not detected using a *S. cerevisiae* expression system with native Hs-*bglA* cDNA.

Therefore, we used a *P. pastoris* expression system with α-factor secretion signal, in which a little enzyme activity was detected in the native Hs-, Sm-, and Rm-*bglA* cDNAs but not in the native Se-*bglA* cDNA. With synthetic (codon-optimized) Se-*bglA* cDNA, a little enzyme activity was detected but the activity was all associated with *P. pastoris* cells and was not secreted.

**Fig. 2.**
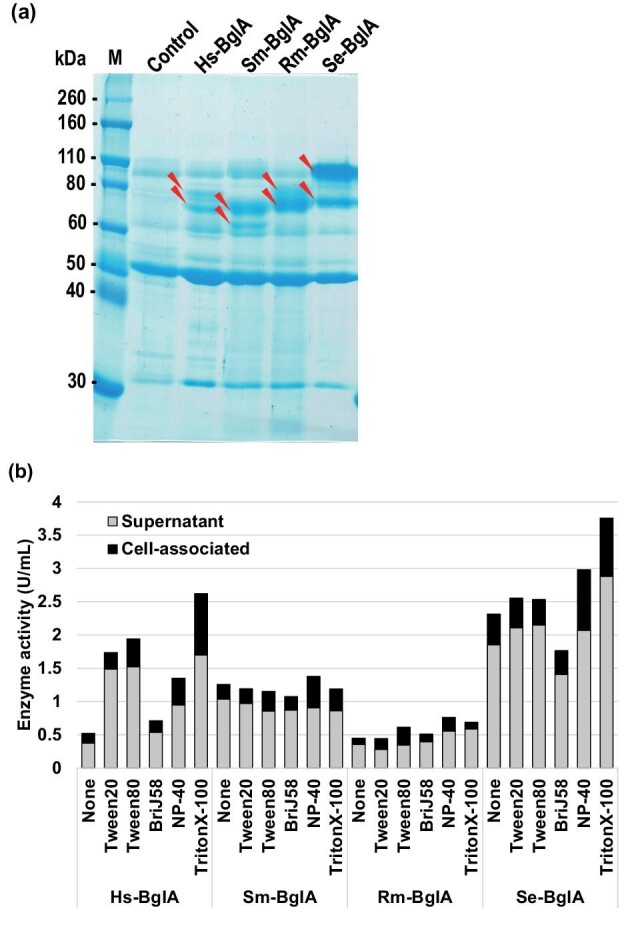
Secreted production of BglA enzymes by recombinant *A. oryzae.* (a) Sodium dodecyl sulfate-polyacrylamide gel electrophoresis (SDS-PAGE) of supernatants. Twenty microliters of each sample was electrophoresed; the β-glycosidase activity in the control, Hs-BglA, Sm-BglA, Rm-BglA, and Se-BglA samples was 0.09, 0.98, 0.40, 0.34, and 1.21 U/mL, respectively. Produced BglA enzymes were entirely in the supernatants. Red arrows indicate putative bands of each BglA enzyme. The molecular weights differed from those calculated because some of them would be processed and/or modified after translation. (b) Effect of nonionic detergents on production of the BglA enzymes. Some nonionic detergents (0.4% [v/v] or [w/v]) increased BglA enzyme production, especially of the Hs- or Se-BglA enzyme. The standard deviation (SD) was omitted because it was within 5% of the mean for all samples.

Considering these results, we tried to produce the BglA enzymes by using codon-optimized *bglA* cDNAs in a filamentous fungi, *A. oryzae*, which has superior protein secretion over that of the yeasts. The recombinant *A. oryzae* produced and secreted the recombinant BglA enzymes, although the Sm-, Rm-, and Se-BglA enzymes appeared as multiple bands, which was caused by heterogeneous glycosylation and/or partial processing (Fig. 2a). At the final sampling point, the β-glycosidase activity in the supernatants was approximately 0.5–2.0 U/mL, which is comparable to the yield of cell-bound β-glycosidase produced by the 2-deoxy-d-glucose-resistant Hs-mutants (Ishikawa et al., 2005) (Fig. 2b). Furthermore, we examined the effect of using nonionic detergents to enhance secretion, but although the increase in total production of Hs- and Se-BglA enzymes was marked, there was little effect on the Sm- and Rm-BglA enzymes (Fig. 2b).

The non-recombinant *A. oryzae* supernatant showed exogenous β-glycosidase activity (0.09 U/mL), but this activity was smaller than that of the BglA enzymes. Consistent with this finding, the non-recombinant *A. oryzae* supernatant produced much less GOS compared with the recombinant *A. oryzae* supernatants. The Se-BglA enzyme could produce GOS up to 90°C, while the Sm-, Rm- and Hs-BglA enzymes were inactivated at 80°C, 80°C and 70°C, respectively (Supplementary Fig. S3). In addition, the BglA enzymes have been biochemically characterized as the native proteins (Blakely & MacKenzie, 1969; Cho et al., 2003; Onishi & Tanaka, 1995, 1996, 1998; Onishi et al., 1995, 1996). For these reasons, we examined the crude BglA enzymes prepared from the supernatants, which would be preferable for use in industrial GOS processes.

### Heat Tolerance and pH Dependency of BglA Enzymes

**Fig. 3.**
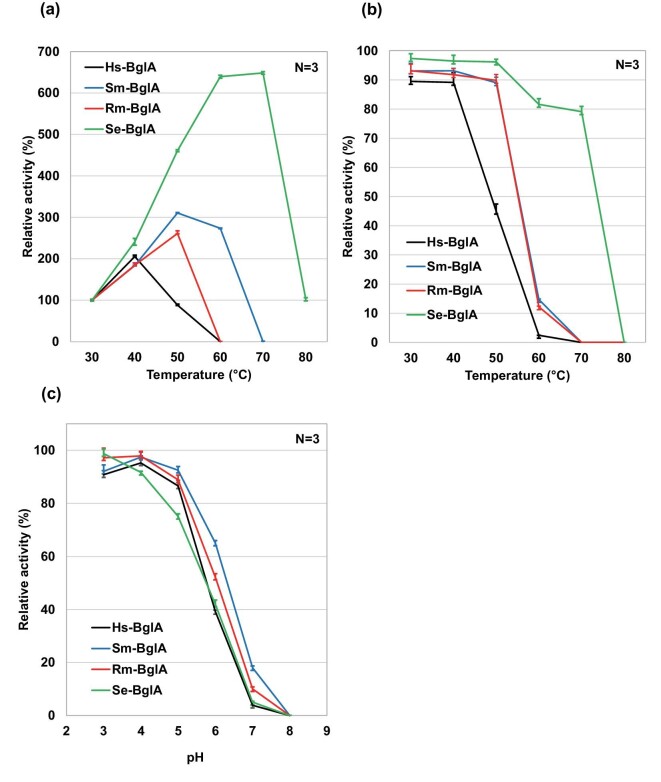
Properties of enzyme concentrates prepared from supernatants. Data shown in (a), (b), and (c) are means of three replicates ± SD. (a) Optimal temperature. Values are expressed as relative activity to that at the standard temperature of 30°C. (b) Thermal stability. Crude enzymes were heated for 30 min before the assay. Values are expressed as relative activity to the sample without heat treatment. (c) Optimal pH. Values are expressed as activity relative to that in the standard condition of 50 mM phosphate citrate buffer (pH 4.0).

We examined the effects of temperature and pH on the crude enzyme concentrates prepared from the Hs-, Sm-, Rm-, and Se-BglA enzymes. The optimal temperature for enzyme activity was 40°C for the Hs-BglA enzyme, 50°C for the Sm- or Rm-BglA enzymes, and 70°C for the Se-BglA enzyme (Fig. 3a). As for the heat stability, after heating for 30 min at the optimal temperature for each enzyme, more than half of the initial activity remained (Fig. 3b). The results show that of the BglA enzymes, the Se-BglA enzyme has remarkable thermostability, which is consistent with the thermostability of the native enzymes (Onishi & Tanaka, 1995, 1996).

The BglA enzymes were more active at acidic pH, and the dependency was similar, but the optimal pH of the Se-BglA enzyme was slightly lower than those of the other BglA enzymes (Fig. 3c).

### Comparison of GOS Production Profiles

We used the crude BglA enzyme concentrates to compare the profiles of GOS production. With the Hs-BglA enzyme, trisaccharide production did not reach a plateau before the endpoint (Supplementary Fig. S4a). At the endpoint, disaccharides containing substrate lactose decreased to half of the initial level and were still decreasing. The Hs-BglA enzyme catalyzed production of relatively fewer tetrasaccharides or longer forms; consequently, the final yield of GOS longer than disaccharides was moderate (Table 4).

**Table 4.** Comparison of GOS Isomers Produced by Basidiomycetous BglA Enzymes

Length	Isomer	*Hamamotoa singularis*	*Sirobasidium magnum*	*Rhodotorula minuta*	*Sterigmatomyces elviae*
Monosaccharides	Gal	3.89	2.14	3.98	2.97
	Glc	20.93	22.05	18.35	21.25
	Total	24.8	24.2	22.3	24.2
Disaccharides	Galβ1-4Glc	25.46	9.53	35.83	6.50
	Galβ1-3Glc	3.14	8.21	1.44	6.15
	Galβ1-2Glc	2.41	8.71	1.40	7.46
	Galβ1-6Glc	3.51	3.13	2.48	10.04
	Others	3.10	2.18	3.87	2.40
	Total	37.6	31.8	45.0	32.6
Trisaccharides	Galβ1-4(Galβ1-6)Glc	0.59	0.57	0.81	0.51
	Galβ1-6Galβ1-4Glc	11.90	6.33	14.49	4.22
	Galβ1-4Galβ1-4Glc	5.62	6.13	2.51	9.75
	Galβ1-4Galβ1-3Glc	0.67	2.95	0.05	3.44
	Others	6.13	10.86	4.50	11.62
	Total	24.9	26.8	22.4	29.5
Tetrasaccharides	Galβ1-6Galβ1-4Galβ1-4Glc	3.72	1.75	1.91	1.41
	Galβ1-4Galβ1-4Galβ1-4Glc	0.84	3.02	0.31	2.77
	Galβ1-6Galβ1-4Galβ1-3Glc	1.32	1.40	0.54	0.93
	Galβ1-4Galβ1-4Galβ1-3Glc	0.76	1.74	0.87	1.85
	Others	3.65	6.26	4.63	5.12
	Total	10.3	14.2	8.3	12.1
Pentasaccharides or longer		2.4	3.0	2.0	1.6
Non-digestible components^a^		49.7	66.3	41.8	69.3
GOS yield to lactose consumed^b^		66.7	73.3	65.1	74.1

Each value represents weight percentage of isomers in solid GOS produced.

^a^Total sum of components except for lactose, glucose, and galactose.

^b^Non-digestive components/(100 – [Galβ1-4Glc]).

With Sm-BglA enzyme, trisaccharide production reached a plateau at 20 h and the decrease of disaccharides slowed after 24 h, with the final disaccharide content below 300 g/L (Supplementary Fig. S4b). The Sm-BglA enzyme catalyzed production of relatively more tetrasaccharides or longer forms, so the GOS yield was high at the endpoint.

Trisaccharide production from the Rm-BglA enzyme was slowest (Supplementary Fig. S4c). Disaccharides containing substrate lactose decreased slowly and more than 400 g/L of disaccharides remained at the endpoint. The Rm-BglA enzyme catalyzed production of relatively fewer tetrasaccharides or larger forms, and among the BglA enzymes, its final GOS yield was lowest.

The Se-BglA enzyme showed a GOS production profile similar to that of the Sm-BglA enzyme (Supplementary Fig. S4d). However, the GOS produced by the Se-BglA enzyme contained less tetra- or longer saccharides and the GOS yield was higher at the endpoint compared with the production by the Sm-BglA enzyme.

### Composition of GOS Isomers

To explore the enzymatic characteristics of each BglA, isomers of disaccharides, trisaccharides, and tetrasaccharides were investigated at the final sampling point of each GOS (Table 4). Among monosaccharides, glucose content was around 20%, but the Rm-GOS contained relatively less glucose. Each GOS sample contained little galactose, suggesting that each BglA enzyme has mainly transgalactosylation activity.

As for disaccharides, there was relatively more residual lactose in the Hs- and Rm-GOS, as represented by the higher disaccharide content seen in Supplementary Fig. S4, whereas there was relatively less in the Sm- or Se-GOS. Furthermore, β-1,3- and β-1,2-translated disaccharides were abundant in the Sm-GOS and β-1,3-, β-1,2-, and β-1,6-translated disaccharides were abundant in the Se-GOS.

As for trisaccharides, Galβ1-6Galβ1-4Glc was a main product in the Hs- and Rm-GOS. The Se-GOS contained relatively more Galβ1-4Galβ1-4Glc, and both Sm- and Se-GOS contained large amounts of other trisaccharides, which presumably have β-1, 3- or β-1, 2- bond considering disaccharide isomer contents in the Sm- or Se-GOS. But we could not identified them because the standards were unavailable.

The tetrasaccharide content in the GOS samples from the four BglA enzymes was not so different, but the Sm- or Se-GOS contained relatively more tetrasaccharides (Supplementary Fig. S4). Notably, Galβ1-4Galβ1-4Galβ1-4Glc content was higher in the Sm-GOS and Se-GOS than in the Hs-GOS and Rm-GOS.

## Discussion

### Features of Basidiomycetous *bglA* Genes and BglA Enzymes

We newly identified three bisidiomycetous *bglA* genes in addition to the Hs-*bglA* gene reported previously (Ishikawa et al., 2005). Many introns, several microexons, and an irregular exon-intron border were identified, similar to those seen in the Hs-*bglA* gene (Table 3), indicating that these are putatively common features of basidiomycetous yeast genes. However, the Se-*bglA* gene contained relatively fewer introns compared with the other *bglA* genes; therefore, details such as intron density may vary with genus, species, or both.

Although all the BglA enzymes belong to the GH1 family, the similarity between their amino acid sequences ranged from 43% to 60%, suggesting that these enzymes constitute a diverse protein family. Additionally, the BglA enzymes commonly had unique N-terminal domains, each either 110 or 210 amino acids long. The Hs-N-terminal domain was reported to contain a non-classical signal sequence addition to a classical peptide (Dagher & Bruno-Barcena, 2016). Using the *P. pastoris* expression system, we examined several deletion mutants of the BglA enzymes and discovered that the N-terminal domain or C-terminal residues were critical to activity (data not shown). Therefore, random deletions would appear to be unusable for releasing active BglA enzymes from the host cells.

### Importance of Hosts for Secreted Production

In this study, the host (i.e. *A. oryzae*) was very important for secreted production, because in *P. pastoris* the BglA enzymes were produced but not secreted and consequently were associated with the cells. Although the mechanism remains unclear, we speculate that the BglA enzymes may contain the motif(s) promoting cell-association in *P. pastoris*, and that the cell-association system does not work in the filamentous fungi *A. oryzae*.

Although native *bglA* cDNAs were not examined in *A. oryzae*, codon optimization would be effective for increasing production (Fleissner & Dersch, 2010; Sasaguri et al., 2008; Tanaka et al., 2012, 2014; Tokuoka et al., 2008; Zhao et al., 2014). Indeed, in the *P. pastoris* expression system we could produce the Se-BglA enzyme only by codon-optimized cDNA, not by the native Se-*bglA* cDNA (data not shown).

### Advantages of Thermostable Enzymes for GOS Production

Although the non-recombinant *A. oryzae* supernatant showed exogenous β-glycosidase activity, it produced little GOS (Supplementary Fig. S3), indicating that the BglA enzymes were responsible for GOS production. Of the BglA enzymes, Se-BglA enzyme showed remarkable thermostability (∼70°C), even though it originated from a mesophilic yeast (Se). These observations are in good agreement with our current understanding of native proteins (Onishi & Tanaka, 1995, 1996). In a GOS reaction mixture with high sugar concentration, the Se-BglA enzyme was able to produce GOS at temperatures up to 90°C, whereas the Sm-, Rm- and Hs-BglA enzymes were inactivated at 80°C, 80°C, and 70°C, respectively (Supplementary Fig. S3). Presumably, sugar concentrations as high as 67% (w/v) in the GOS reaction mixture would protect the BglA enzymes against heat denaturation. This thermostability suggests that the Se-BglA enzyme may have several advantages for industrial processes. For example, high temperatures (∼90°C) could be used to selectively denature proteins produced by *A. oryzae* while retaining the activity of the Se-BglA enzyme. The thermotolerance of proteins produced by *A. oryzae* with respect to β-glycosidase activity was almost the same as that of the Hs-BglA enzyme; therefore, undesired products of reactions catalyzed by contaminants produced by *A. oryzae* would be eliminated at temperatures above 70°C. In addition, the higher temperature would prevent bacterial contamination and lactose crystallization, and would accelerate GOS production. Moreover, the time taken to cool to the reaction temperature after dissolving lactose would be shortened.

Except for lactose, glucose, and galactose, which are absorbed and metabolized by the epithelial cells of the gastrointestinal tract, the active components of GOS are regarded as non-digestible. The non-digestible components were calculated to be 69.3% in Se-GOS, 66.3% in Sm-GOS, 49.7% in Hs-GOS, and 41.8% in Rm-GOS. In addition, the percentage GOS yield to lactose consumed was calculated to be 74.1% in Se-GOS, 73.3% in Sm-GOS, 66.7% in Hs-GOS, and 65.1% in Rm-GOS (Table 4). Consequently, the Se-BglA enzyme was the most promising enzyme in terms of thermostability, non-digestible GOS content, and GOS yield, although the Sm-BglA enzyme showed similar results.

### Features of GOS Constituents

Although the amount of each BglA enzyme used for GOS production was adjusted so that the amount of ONPG hydrolysis activity was the same, the speed of GOS production for each enzyme was different (Supplementary Fig. S4). Thus, the ratio of GOS productivity to ONPG hydrolysis activity might differ among BglA enzymes. Besides, each enzyme produced different amounts of GOS longer than trisaccharides (Supplementary Fig. S4), and concomitantly their bond preference (β-1,4-, β-1,6-, β-1,3-, and β-1,2-) was also distinctive (Table 4). For example, the Se-BglA enzyme produced low Gal1-6Gal1-4Glc and high Gal1-6Glc, indicating that Se-BglA prefers glucose over lactose as the acceptor for generating β-1,6-bond. Such preferences likely originate from the arrangement of subsites in each BglA enzyme (Qin et al., 2019). A previous report has shown that the catalytic properties of a *Bacillus circulans* β-galactosidase can be successfully modulated by binding artificial proteins, termed monobodies, to the subsites (Tanaka et al., 2015). At present, more detailed structural studies would be necessary to unveil such subsites. The composition of GOS produced by the BglA enzymes and the crystal structures of the Hs-BglA enzyme have been reported, which together provide data on the putative function of the unique N-terminal domain and subsites of the Hs-BglA enzyme (Uehara et al., 2020). Further structural information would be helpful to investigate the mechanism that gives the Se-BglA enzyme its remarkable thermostability.

By using the available structural information and characterizations of the BglA enzymes, in the future we hope to be able to use protein engineering to rearrange the enzyme subsites to obtain GOS isomer compositions tailored for specific targets (e.g. infants, adolescents, and beneficial microbes).

## Supplementary Material

kuab087_Supplemental_File
