# Supplementary material for: Molecular characterization and secreted production of basidiomycetous cell-bound β-glycosidases applicable to production of galactooligosaccharides
Source: J Ind Microbiol Biotechnol. 2021 Dec 8;49(3):kuab087. doi: 10.1093/jimb/kuab087 (PMC9142197; doi:10.1093/jimb/kuab087)
Supplement: kuab087_Supplemental_File [file kuab087_supplemental_file.pdf]

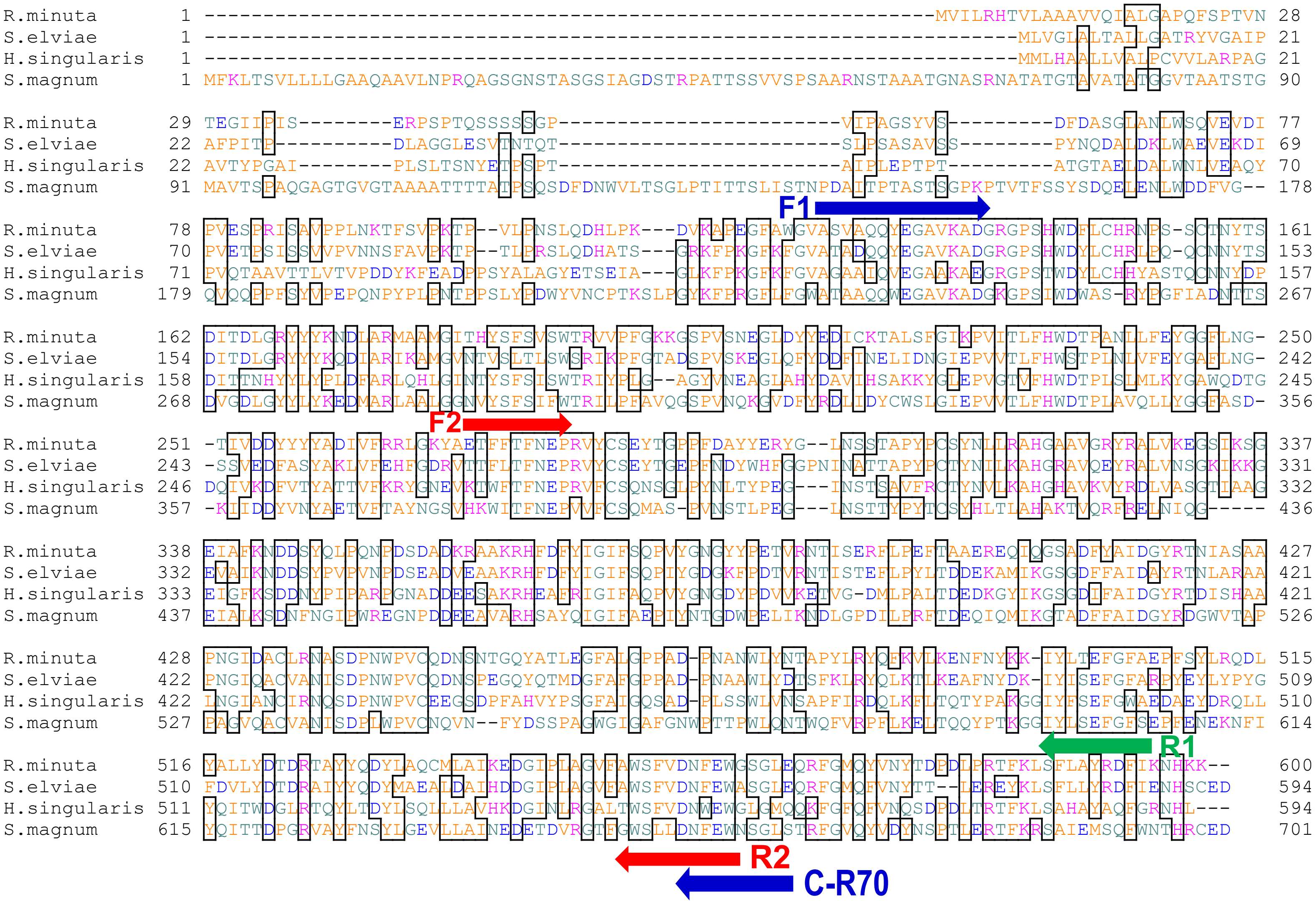

FIG S1

Multiple sequence alignment of basidiomycetous BglA enzymes and degenerated primers

ClustalW2 was used to compare homologous sequences so that putative catalytic centers were identified.

Annealing sites of the degenerated primers in Table 1 are indicated with arrows, and the motifs or putatively conserved residues are boxed.

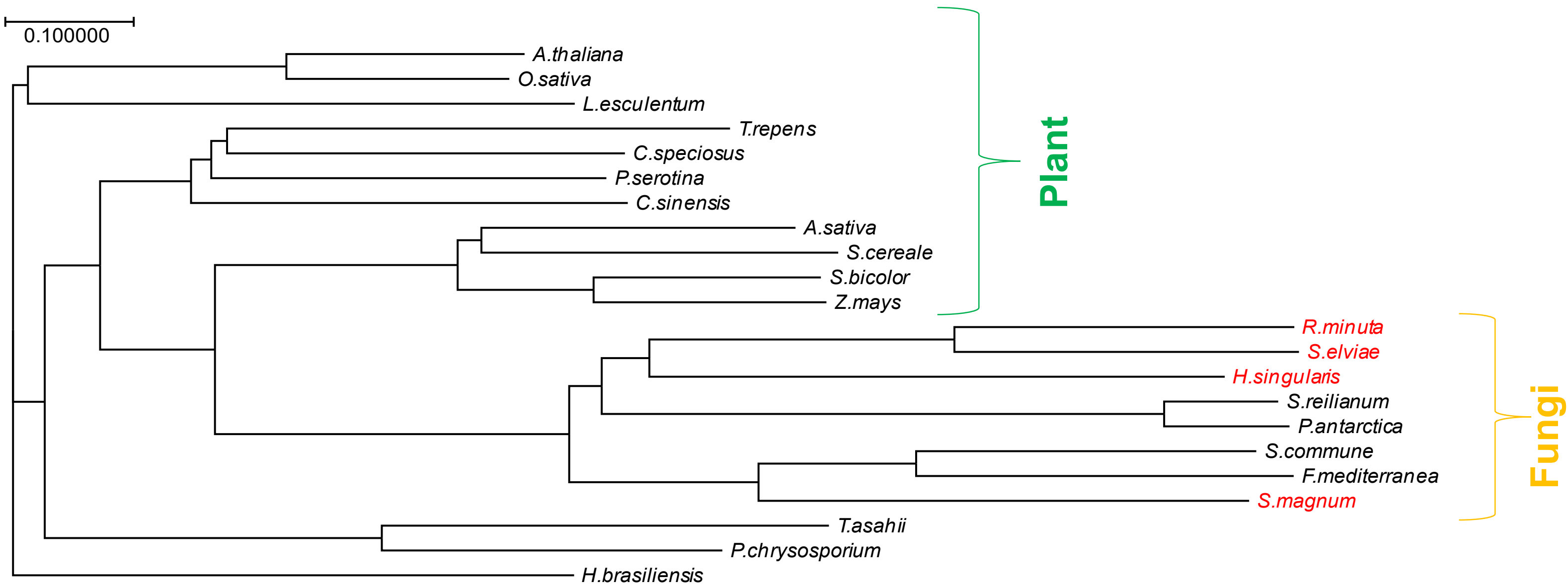

FIG S2

Phylogenetic tree of plant β-glucosidases, non-basidiomycetous fungal β-glucosidases, and basidiomycetous (red) BglA enzymes

ClustalW2 was used to analyze several β-glucosidases found in GH1 based on their amino acid sequences. *A. sativa: Avena sativa* / AF082991; *A. thaliana: Arabidopsis thaliana* / AY142610; *C. sinesis: Camellia sinensis* / AB088027; *C. speciosus: Costus speciosus* / D83177; *F. mediterranea : Fomitiporia mediterranea* / R7SG13; *H. singularis: Hamamotoa singularis* (previous study) / AB126324; *H. brasiliensis: Hevea brasiliensis* / AY297039; *L. esculentum: Lycopersicon esculentum* / AF403444; *O. sativa: Oryza sativa* / AK071372; *P. chrysosporium: Phanero-chaete chrysosporium* / Q25BW4; *P. serotina: Prunus serotina* / U50201; *P. antarctica: Pseudozyma antarctica* / M9M087; *R. minuta: Rhodotrula minuta* (this study); *S. commune: Schizophyllum commune* / D8PVX2; *S. cereale: Secale cereale* / AF293849; *S. magnum: Sirobasidium magnum* (this study); *S. bicolor: Sorghum bicolor* / U33817; *S. reilianum: Sporisorium reilianum* / E7A3C8; *S. elviae Sterigmatomyces elviae* (this study); *T. asahii: Trichosporon asahii* / K1VV22; *T. repens: Trifolium repens* / X56734; *Z. mays: Zea mays* / X74217. Code numbers are the accession numbers in the DDBJ/GENBANK/EMBL nucleotide databanks.

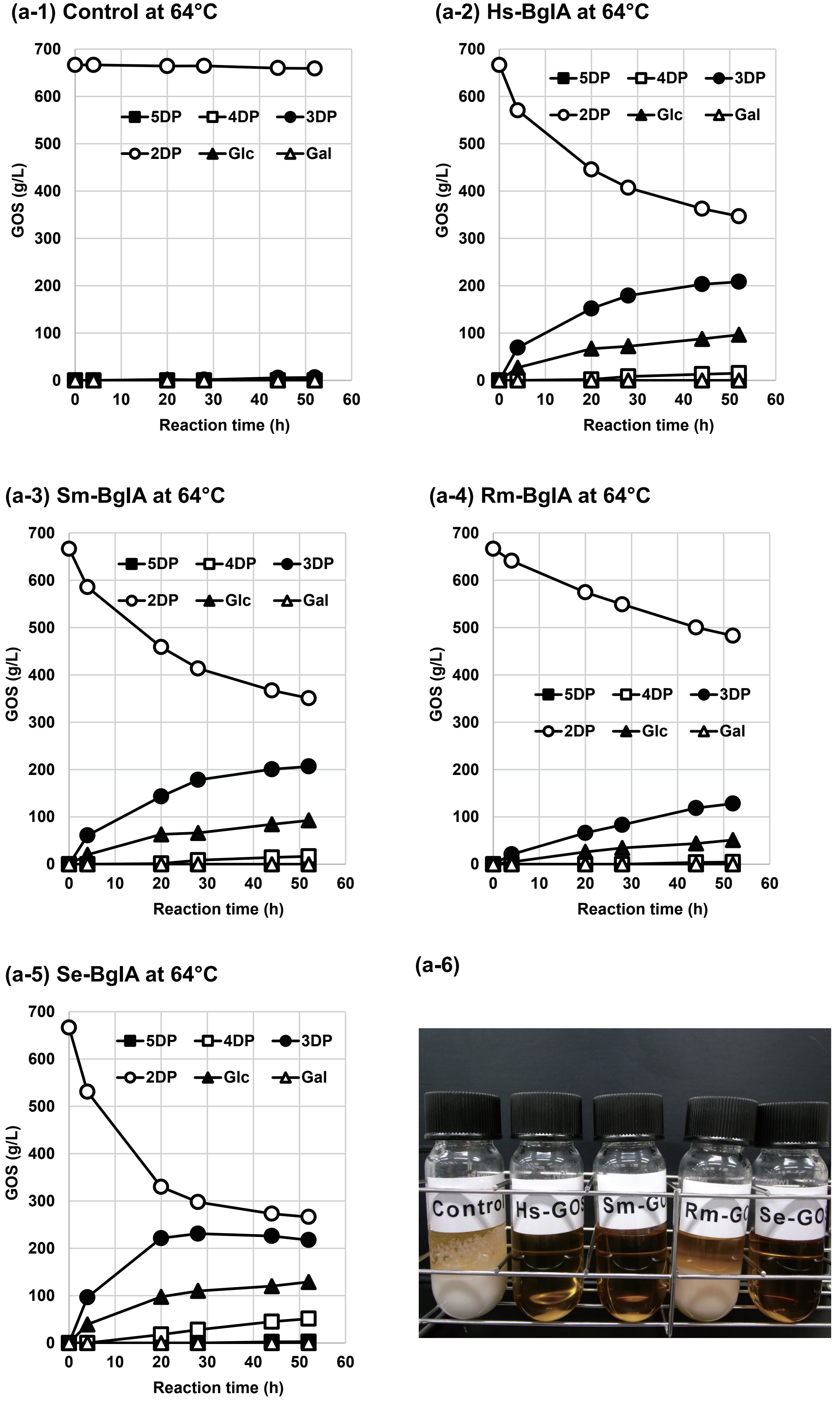

FIG S3
Effects of temperature on galactooligosaccharide (GOS) production by non-recombinant or recombinant *Aspergillus oryzae* supernatants
Control, non-recombinant *A. oryzae*; Hs, *Hamamotoa singularis*; Sm, *Sirobasidium magnum*; Rm, *Rhodotorula minuta*; Se, *Sterigmatomyces elviae*. Gal, galactose; Glc, glucose; 2DP, disaccharides; 3DP, trisaccharides; 4DP, tetrasaccharides; 5DP, pentasaccharides. A sample (1 mL) of each supernatant was added to 30 mL of 66.7% (w/v) lactose solution (pH 6.0); the β-glycosidase activity in the control, Hs-BglA, Sm-BglA, Rm-BglA, and Se-BglA supernatants was 0.09, 1.70, 0.86, 0.59, and 2.88 U/mL, respectively. The photographs in the figure show the appearance of the GOS reaction mixtures at the final time point. Residual lactose was crystallized at room temperature.

(b-1) Control at 70°C

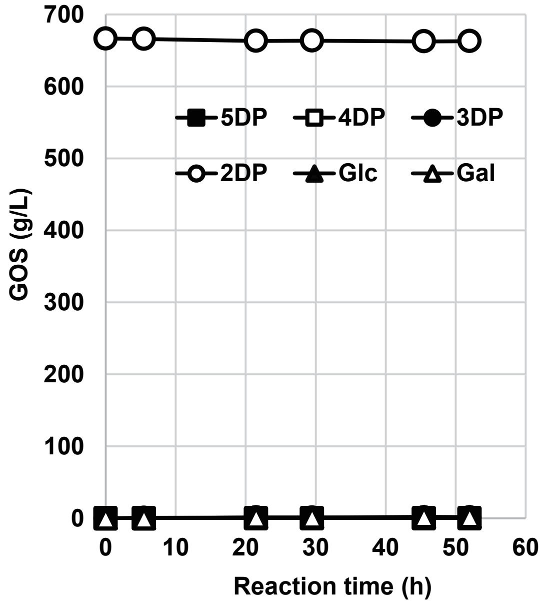

(b-2) Hs-BglA at 70°C

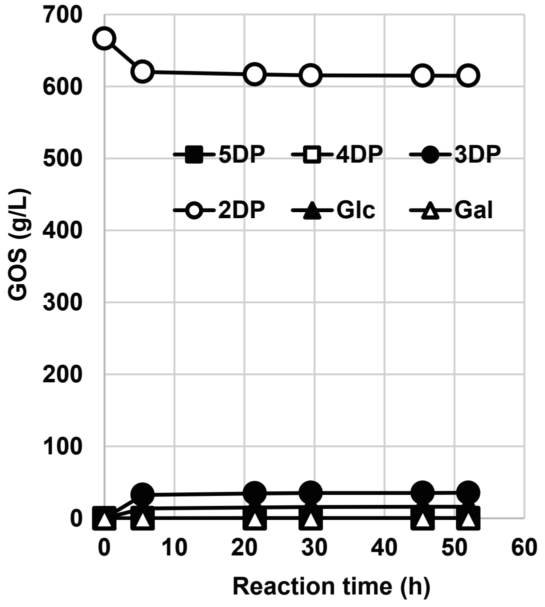

(b-3) Sm-BglA at 70°C

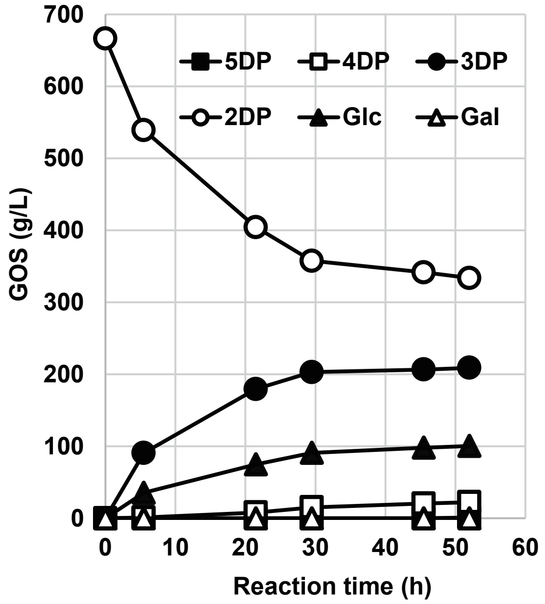

(b-4) Rm-BglA at 70°C

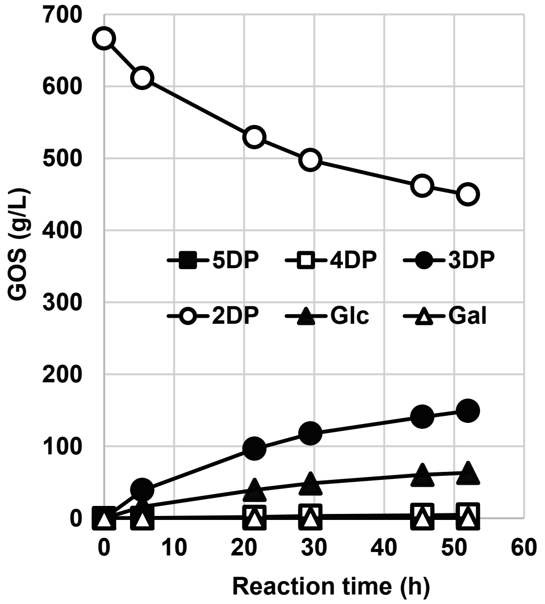

(b-5) Se-BglA at 70°C

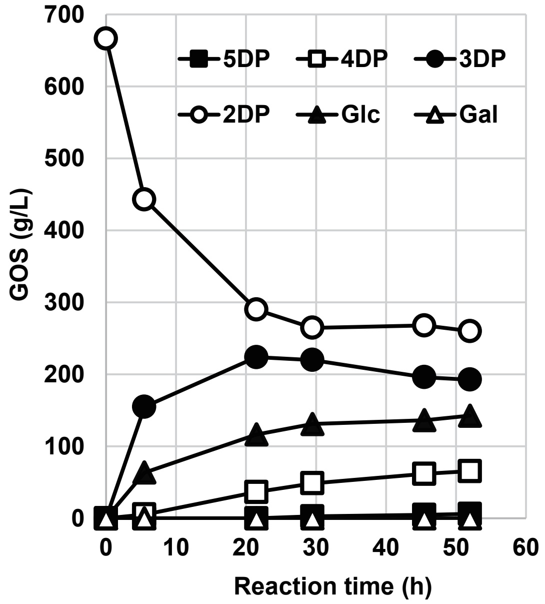

(b-6)

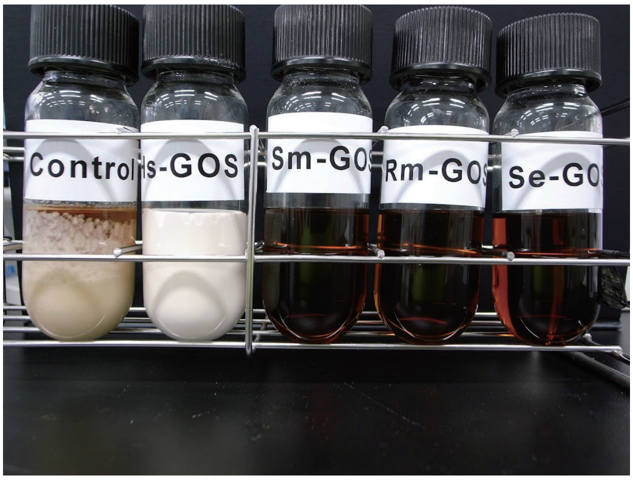

FIG S3
Continued

(c-1) Control at 80°C

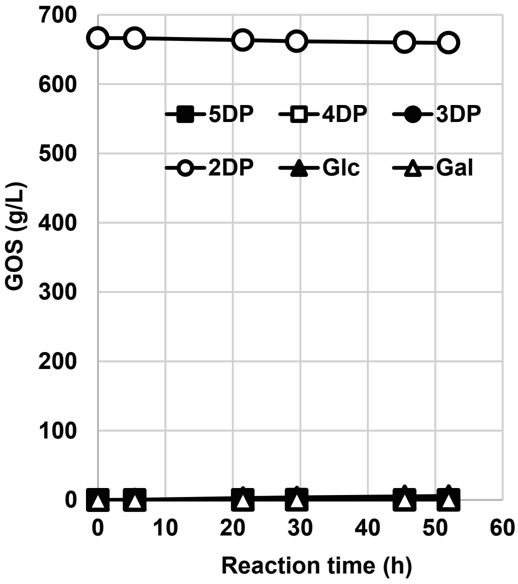

(c-2) Hs-BglA at 80°C

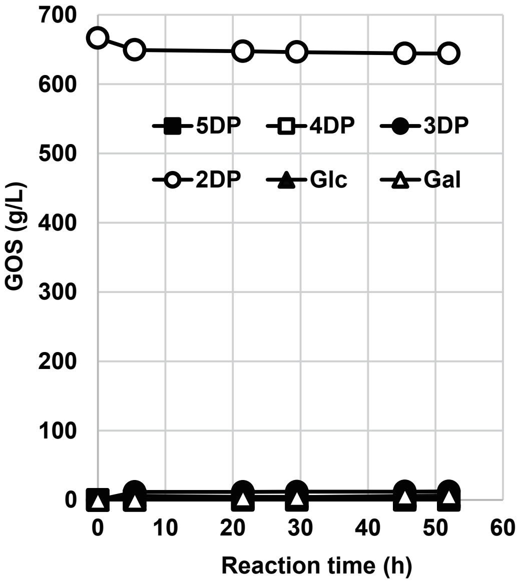

(c-3) Sm-BglA at 80°C

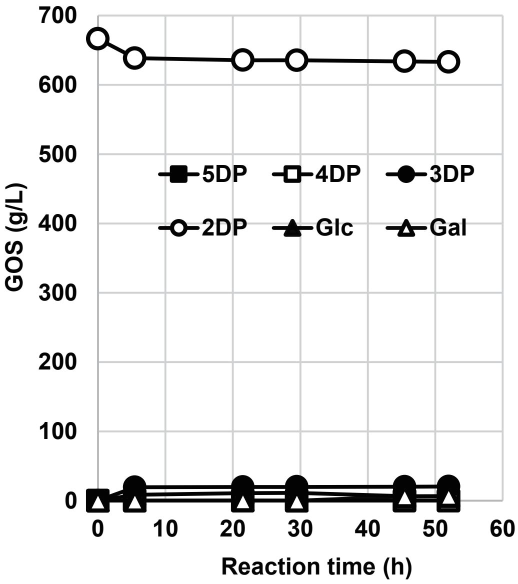

(c-4) Rm-BglA at 80°C

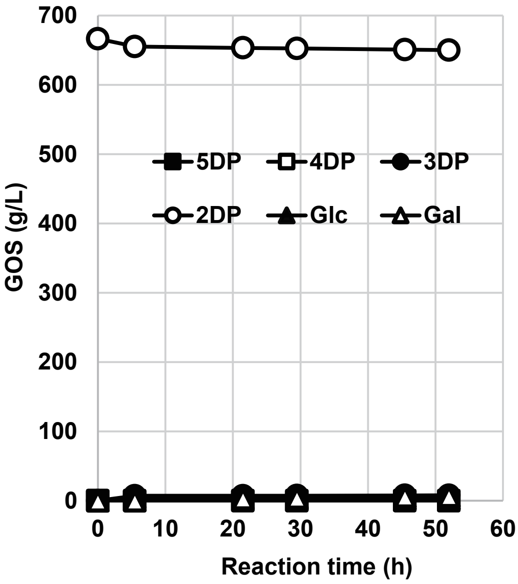

(c-5) Se-BglA at 80°C

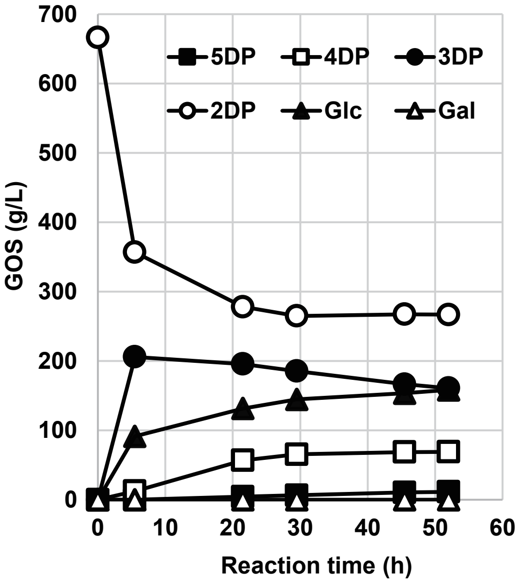

(c-6)

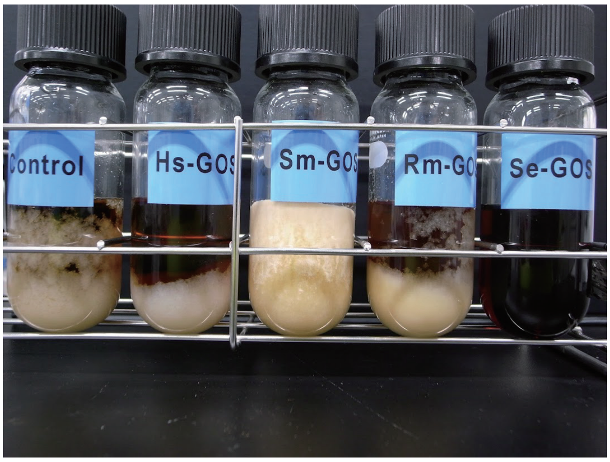

FIG S3
Continued

(d-1) Control at 90°C

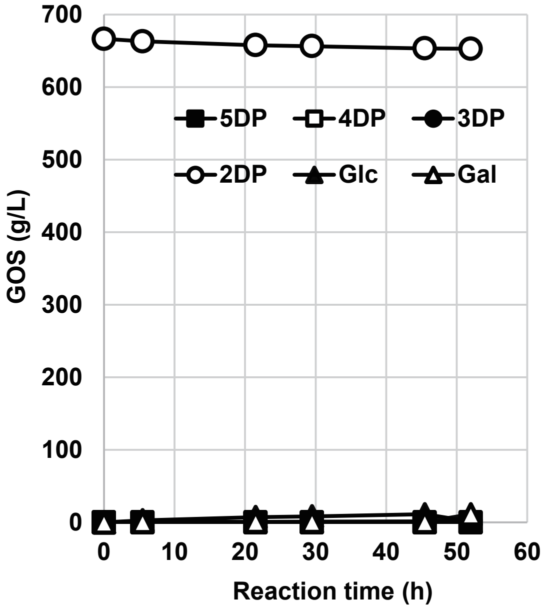

(d-2) Hs-BglA at 90°C

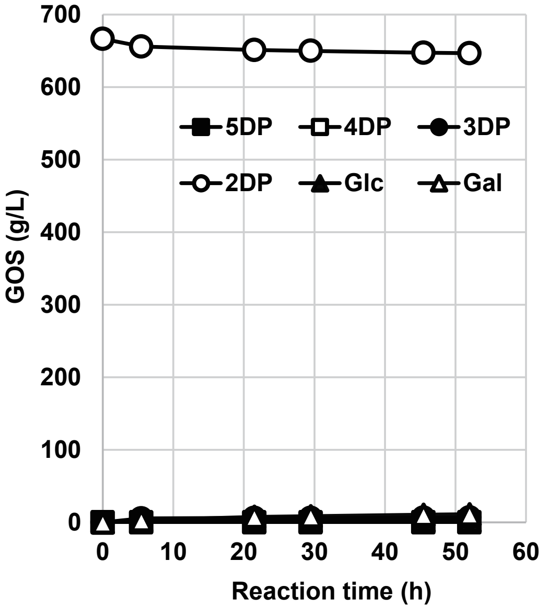

(d-3) Sm-BglA at 90°C

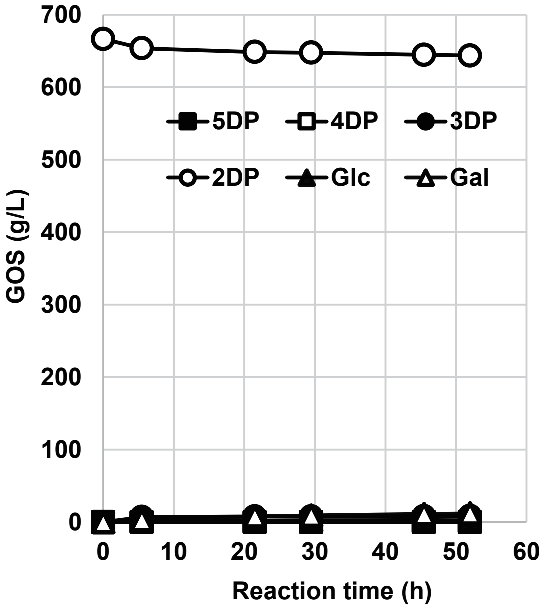

(d-4) Rm-BglA at 90°C

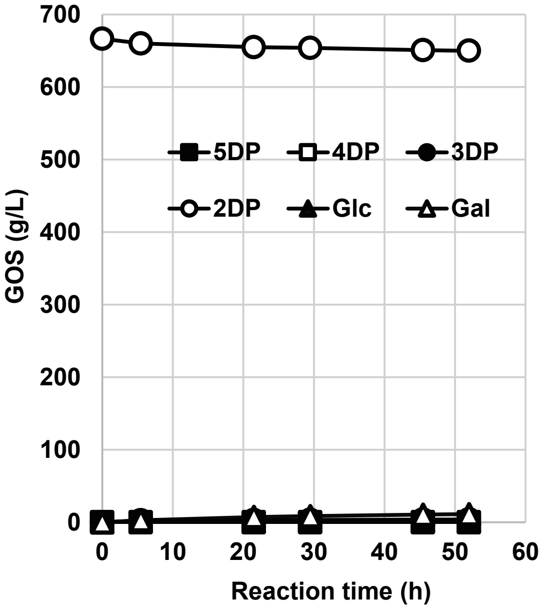

(d-5) Se-BglA at 90°C

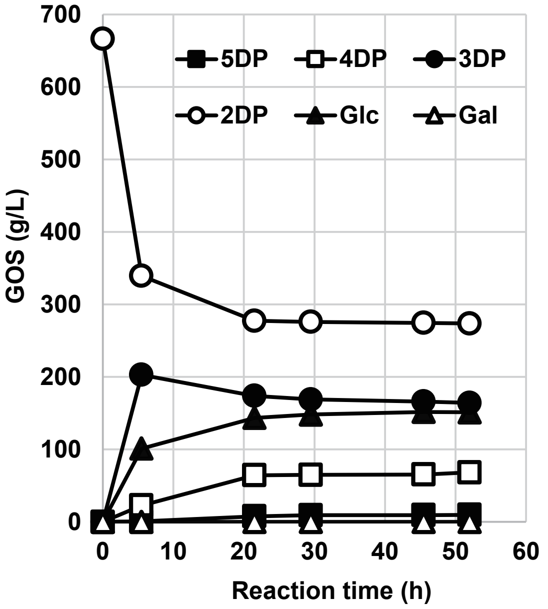

(d-6)

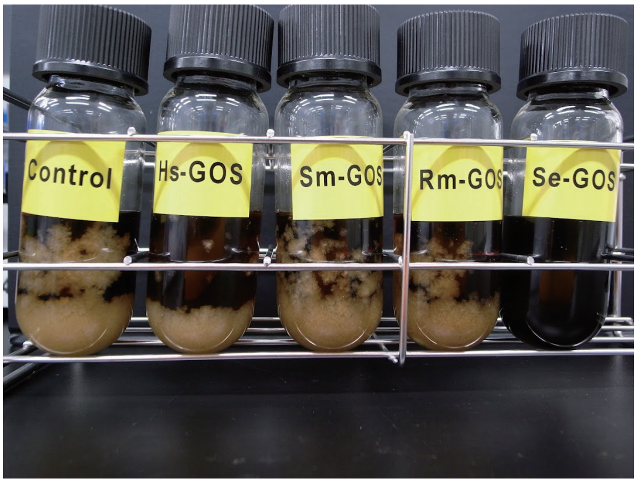

FIG S3
Continued

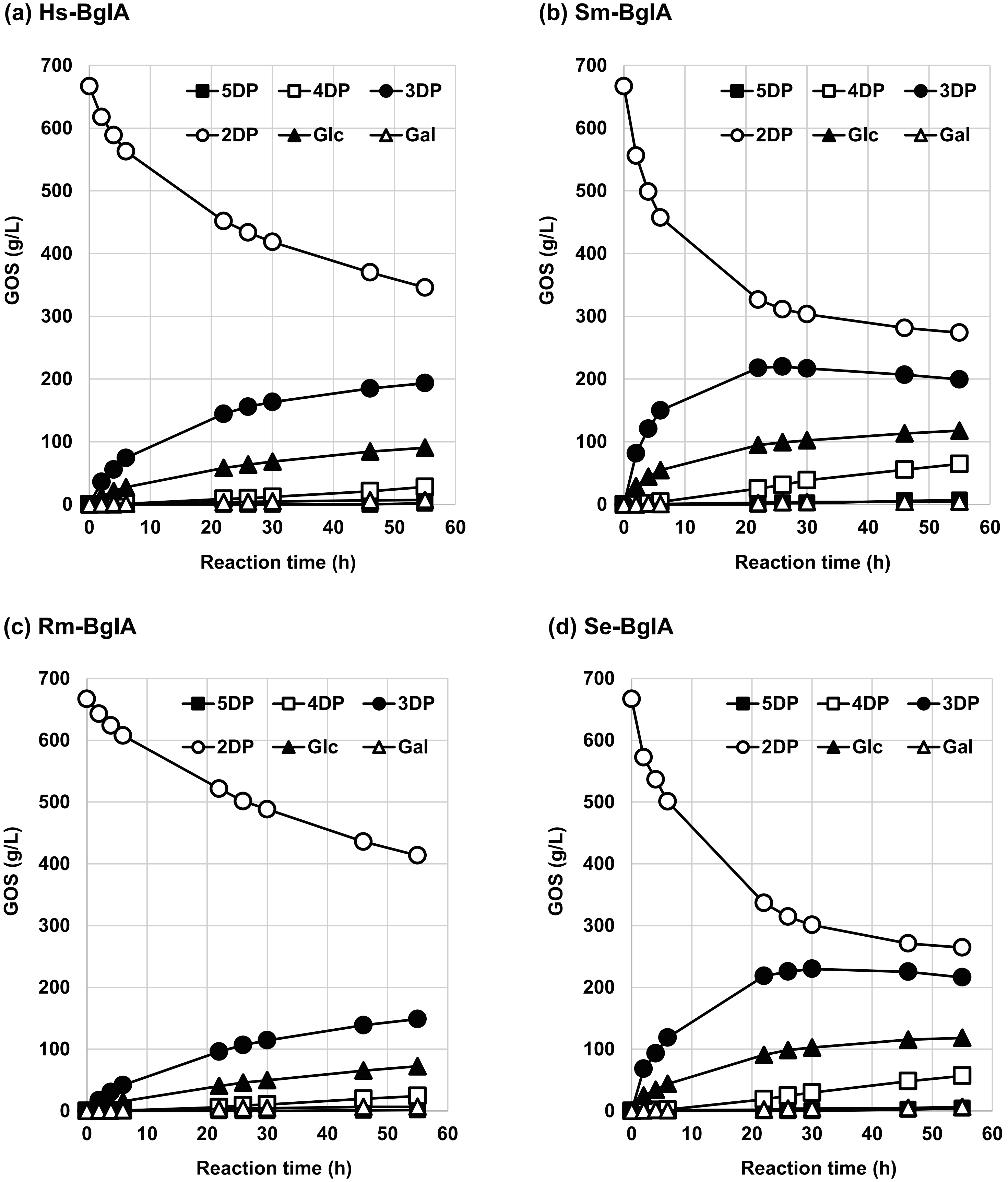

FIG S4

Time course of GOS production by enzyme concentrates at 64 °C

(a) *Hamamotoa singularis* (Hs-BglA); (b) *Sirobasidium magnum* (Sm-BglA); (c) *Rhodotorula minuta* (Rm-BglA); (d) *Sterigmatomyces elviae* (Se-BglA). Gal, galactose; Glc, glucose; 2DP, disaccharides; 3DP, trisaccharides; 4DP, tetrasaccharides; 5DP, pentasaccharides.
